# Modulation of Brain β-Endorphin Concentration by the Specific Part of the Y Chromosome in Mice

**DOI:** 10.1371/journal.pone.0016704

**Published:** 2011-03-08

**Authors:** Michel Botbol, Pierre L. Roubertoux, Michèle Carlier, Séverine Trabado, Sylvie Brailly-Tabard, Fernando Perez-Diaz, Olivier Bonnot, Guillaume Bronsard, Sylvie Tordjman

**Affiliations:** 1 INSERM U 669, Troubles des Conduites Alimentaires à l'Adolescence, Paris, France; 2 Aix Marseille Université, INSERM U 910, Génétique Médicale Génomique Fonctionnelle, Marseille, France; 3 Aix Marseille Université and Institut Universitaire de France, Laboratoire de Psychologie Cognitive, CNRS UMR 6146, Marseille, France; 4 INSERM U 693, Université Paris-Sud, Faculté de Médecine Paris-Sud, Kremlin-Bicêtre, Assistance Publique-Hôpitaux de Paris, CHU Bicêtre, Service de Génétique Moléculaire, Pharmacogénétique et Hormonologie, Le Kremlin-Bicêtre, France; 5 Centre Emotion, UFR 3 Service Hospitalo-Universitaire de Psychiatrie de l'Enfant et de l'Adolescent 246 CNRS, Groupe Hospitalier Pitié-Salpétrière, Paris, France; 6 Service Hospitalo-Universitaire de Psychiatrie de l'Enfant et de l'Adolescent, Hôpital Pitié-Salpêtrière, Paris, France; 7 Laboratoire de Santé Publique (EA3279), Ecole de Médecine de LaTimone, Marseille, France; 8 Service Hospitalo-Universitaire de Psychiatrie de l'Enfant et de l'Adolescent de Rennes, CHGR et Université de Rennes 1, Rennes, France; 9 Laboratoire Psychologie de la Perception, Université Paris Descartes, Paris, France; 10 CNRS UMR 8158, Paris, France; Université Pierre et Marie Curie, France

## Abstract

**Background:**

Several studies in animal models suggest a possible effect of the specific part of the Y-chromosome (*Y^NPAR^*) on brain opioid, and more specifically on brain β-endorphin (BE). In humans, male prevalence is found in autistic disorder in which observation of abnormal peripheral or central BE levels are also reported. This suggests gender differences in BE associated with genetic factors and more precisely with *Y^NPAR^*.

**Methodology/Principal Findings:**

Brain BE levels and plasma testosterone concentrations were measured in two highly inbred strains of mice, NZB/BlNJ (N) and CBA/HGnc (H), and their consomic strains for the *Y^NPAR^*. An indirect effect of the *Y^NPAR^* on brain BE level via plasma testosterone was also tested by studying the correlation between brain BE concentration and plasma testosterone concentration in eleven highly inbred strains. There was a significant and major effect (*P*<0.0001) of the *Y^NPAR^* in interaction with the genetic background on brain BE levels. Effect size calculated using Cohen's procedure was large (56% of the total variance). The variations of BE levels were not correlated with plasma testosterone which was also dependent of the *Y^NPAR^*.

**Conclusions/Significance:**

The contribution of *Y^NPAR^* on brain BE concentration in interaction with the genetic background is the first demonstration of Y-chromosome mediated control of brain opioid. Given that none of the genes encompassed by the Y*^NPAR^* encodes for BE or its precursor, our results suggest a contribution of the sex-determining region (*Sry*, carried by Y*^NPAR^*) to brain BE concentration. Indeed, the transcription of the Melanocortin 2 receptor gene (*Mc2R* gene, identified as the proopiomelanocortin receptor gene) depends on the presence of *Sry* and BE is derived directly from proopiomelanocortin. The results shed light on the sex dependent differences in brain functioning and the role of *Sry* in the BE system might be related to the higher frequency of autistic disorder in males.

## Introduction

The Y chromosome includes the *Y^NPAR^* and the *Y^PAR^*. The *Y^NPAR^* is called non-pairing or specific region and is transmitted from father to sons exclusively. The *Y^PAR^* recombines with the X chromosome at the male meiosis and is called pairing or pseudoautosomal region for this reason. Few functional genes are mapped on *Y^NPAR^* (histocompatibility Y antigen, RNA binding motif protein, and several other genes contributing to male reproduction such as *Sry* and genes necessary for the spermatozoon development and maintenance). Several lines of evidence suggest a possible effect of *Y^NPAR^* on brain opioid, and more specifically on brain β-endorphin (BE). Neonatal injection of testosterone decreases brain BE concentration and the number of μ receptors in the hypothalamus (μ receptors are receptors of BE) [1], [2]. In addition, neonatal injection or exposure to testosterone contributes to the “male pattern” of the ontogenesis of μ receptors in the hypothalamus and to the development of BE innervations in the brain [2]–[6]. Inversely, intracerebro-ventriculaire injection of BE decreases plasma Luteinizing Hormone concentration and consequently plasma testosterone concentration; this effect involves the μ receptors and is blocked by the preliminary administration of Naloxone (an antagonist of the μ receptors) [7]–[10]. Given these previous observations and because *Y^NPAR^* is involved in plasma testosterone concentration and testicular reactivity to testosterone [11], [12], the *Y^NPAR^* is expected to be associated with brain BE.

This hypothesis of an effect of the *Y^NPAR^* on brain BE is also supported by studies in mice models and in humans showing an inhibitory influence of central opioids acting through the μ receptors (such as BE) on aggressive behavior [13]–[20], and an effect of the murine *Y^NPAR^* on aggression [21]–[25]. In addition, Laarakker et al.'s study [26] reporting a contribution of the *Y^NPAR^* to anxiety-related behavior in mice, strengthens the hypothesis of an effect of the *Y^NPAR^* or brain BE, given that BE is considered as a stress hormone [27]–[29].

Finally, in humans, and more precisely in autistic disorder, two other arguments support our hypothesis: on one hand, the fourfold higher prevalence of autism in male compared to female [30] (autism is a pervasive developmental disorder for which family and twin studies suggest a genetic contribution [31]–[33]) could indicate a contribution of the *Y^NPAR^*; on the other hand, several studies have reported abnormal central as well as peripheral BE levels in individuals with autism [34].

The present study was designed first to test directly the effect of *Y^NPAR^* on brain BE levels in two highly inbred strains of mice, NZB/BlNJ (N) and CBA/HGnc (H), and their consomic strains for the *Y^NPAR^*. Second, an indirect effect of the *Y^NPAR^* on brain BE level via plasma testosterone was tested by studying the genetic correlation between brain BE concentration and plasma testosterone concentration in eleven highly inbred strains of mice.

## Methods

### Mice and rearing conditions

Brain β-endorphin and plasma testosterone were measured in a set of inbred strains of mice and in a quartet of parental and their consomic strains for the *Y^NPAR^*. The set of inbred strains consisted in 11 inbred mouse strains A/J, XLII, BA, BALB/cBy, C57BL/10Bg, C57BL/6Jby, CPB-K, DBA/1Bg, DBA/2j, CBA/HGnc and NZB/BlNJ. The strains were maintained in the laboratory for six or more generations of brother-sister mating regimen. The quartet of parental and their consomic strains for the *Y^NPAR^* was developed as follows: we selected two strains of laboratory mice NZB/BlNJ (N) and CBA/HGnc (H) that were known for the different origin of their respective Y chromosomes. Furthermore, N and H were selected because the N males, who present the highest scores for aggression and the highest plasma testosterone concentration compared to the H males [1], [3], [7]–[10], [13]–[20], were expected to have also lower brain BE levels as suggested by previous reports described in the introduction [13], [35]. The production of the consomics for the *Y^NPAR^* has been previously described [12], [36]. The HNF1 males carried the N-*Y^NPAR^* of the N donor strain because the *Y^NPAR^* is paternally transmitted. The males were sired with an H female. The progeny carried the N-*Y^NPAR^* but 50% of the N genes were lost and replaced by H genes at each generation. The N-*Y^NPAR^* was substituted to the H-*Y^NPAR^* after several generations of repeated backcrosses. The H strain and its consomic H.N-*Y^NPAR^* differed only by the origin of N-*Y ^NPAR^* that is H-*Y^NPAR^* in the parental and N-*Y^NPAR^* in the consomics. A similar strategy was developed to develop the consomic N.H- *Y^NPAR^*, with the N strain as recipient and H as donor strain for the *Y^NPAR^*. The males came from the 25 and 26^th^ backcross generations when they were used for the present experiments. The probability to have one allele from the autosomes, X chromosome and *Y^PAR^* from the donor strain in the consomic is below 5×10^−8^ under these conditions. In addition, with homozygous autosomal alleles no genomic imprinting could contribute to the difference between a parental and its congenic strain. The isogenicity between each parental and its consomic strain was confirmed by the acceptance of reciprocal skin grafts and identity of mandible shape measurements [37], [38] because both are controlled by a large number of loci spread on the genome [39]. This isogenicity between each parental and the respective consomic strain was also tested by comparing 123 *Mit* polymorphisms (unpublished). Moreover, each parental strain and its consomic had identical genetic information for the *Sts* locus mapped on the *Y^PAR^* [36].

Four to six littermates were housed with their mother in a 42×11×18 cm plastic cage and a bedding of dust-free sawdust with food and water ad libitum, temperature 24.0±0.5°C and lights on at 08:00 AM. Weaning took place at 28±2 days of age. Males were then placed with the same age female (usually a littermate) and were killed at 66±4 days by cervical dislocation. Cervical dislocation was selected as being the more rapid technique for euthanasia. The whole brains (after removing olfactory bulbs and cerebellum) were saved for biochemical analyses and stored at −80°C. The blood was collected by intra-cardiac puncture in 10 µl of heparin and the plasma was kept at −25°C. The experimental protocol with the mice was conducted according to the European guidelines and was approved by the National Committee of the Scientific Research (CNRS).

### Biochemical analyses

Brain tissues were homogenized in 5 volumes of 0.1 *M* HCl and heated for 15 min at 95°C. After centrifugation (38,000×*g*, 10 min, 4°C), the supernatant was adjusted to pH 7.0 with 1 *M* Tris base, and the resulting precipitate was spun down at 6,000×*g* for 10 min at 4°C. Protein concentrations were determined in the pellets by the method of Lowry et al. [40] with bovine serum albumin as the standard. Brain BE levels are expressed in picomoles per gram of fresh tissue.

The plasma testosterone was assayed according to RIA in coated tubes using a specific testosterone antibody exhibiting a reduced cross-reaction (7.5%) with other androgen steroids (^125^ I-TESTOSTERONE COATRIA, kit Biomérieux). The competitive inhibition reaction was measured between a fixed amount of ^125^ I-labelled testosterone and the testosterone to be determined (standards or samples) for a fixed number of binding sites on the anti-testosterone antibody. Coated tubes, containing plasma and ^125^ I-testosterone tracer, were incubated for 2 h at 37°C. The ^125^ I-testosterone bound was separated from that remaining in solution. The bound radioactivity counted for 1 min was then inversely proportional to the testosterone quantity in standards or samples. Each individual sample was assayed in duplicate and the sample again assayed when the difference between the two results was over 20%.

### Statistical analyses

The effect of the *Y^NPAR^* on brain BE level and plasma testosterone concentration was studied using a two-way ANOVA, with genetic background -H versus N- and origin of the *Y^NPAR^* -H versus N- as main factors. Effect size was calculated using the *θ^2^* statistic and expressed as a percentage of variance [41]. Comparisons between the 11 inbred mouse strains for BE or testosterone variables were performed using ANOVA. The Kolmogorov-Smirnov test indicated that BE and testosterone levels were not normally distributed; thus all ANOVAs were performed using log-transformed BE and testosterone values. Correlations between brain BE levels and plasma testosterone concentrations in the 11 inbred mouse strains (each strain is represented by the mean of five animals for biological variables) were determined by Spearman rank-order correlation analyses. According to Hegmann and Possidente [42], these correlations between biological measures can be considered as an estimation of genetic correlations. The significance level was set at 0.05; however, the usual level of 0.05 is very conservative when the correlations are computed on mean scores and not on individual scores.

## Results

### Effect of the Y^NPAR^ on brain β-endorphin levels

**Figure 1:**
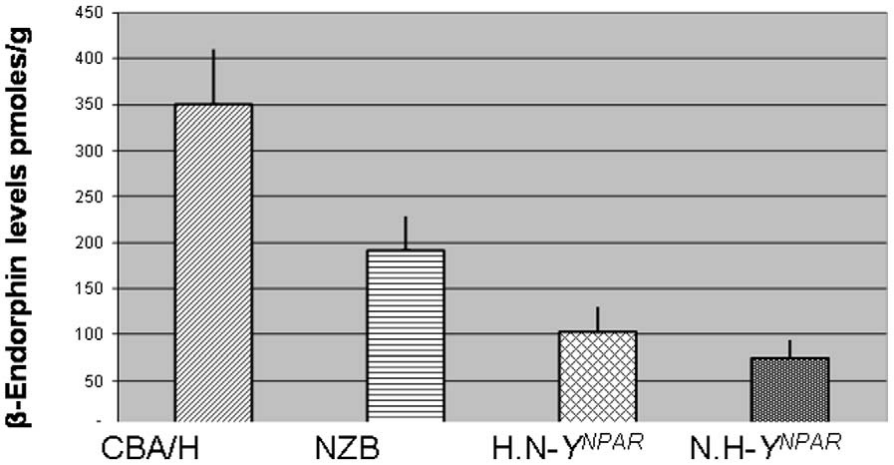
Brain BE concentration (mean ±SEM) in NZB and CBA/H and their consomic strains for *Y^NPAR^*. The N.H-*Y^NPAR^* differs only from the NZB by the *Y^NPAR^* from CBA/H, and the H.N- *Y^NPAR^* differs only from the CBA/H by the *Y^NPAR^* from NZB. Partial comparisons with Student's t test showed that the parental NZB and CBA/H strains differed significantly (*P*<0.05), and each parental strain differed significantly from its consomic strain (CBA/H *vs.*H.N-*Y^NPAR^*: *P*<0.001; NZB *vs.* N.H-*Y^NPAR^*: *P*<0.01); *n* = 5 animals for each strain, except for NZB (*n* = 6); *SEM* = standard error of the mean.

Brain BE levels were significantly modified by the non-*Y^NPAR^* genotype and the *Y^NPAR^* in interaction with non- *Y^NPAR^* genotype (F(1,17) = 4.80, *P*<0.04, *θ^2^* = 0.10 and F(1,17) = 27.18, *P*<0.0001, *θ^2^* = 0.56, respectively). The *Y^NPAR^* alone did not contribute significantly to BE concentration (F(1,17)<1). Partial comparisons indicated that BE levels were significantly higher in the H strain than in the N strain (t (1.47) = 2.43, *P*<0.05). The substitution of the *Y^NPAR^* from H by the *Y^NPAR^* from N, in the H strain, reduced significantly the brain BE concentration, and the substitution of the *Y^NPAR^* from N by the *Y^NPAR^* from H, in the N strain, reduced the brain BE level to the lowest concentration.(see **Figure 1**).

### Effect of the Y^NPAR^ on plasma testosterone concentration

**Figure 2:**
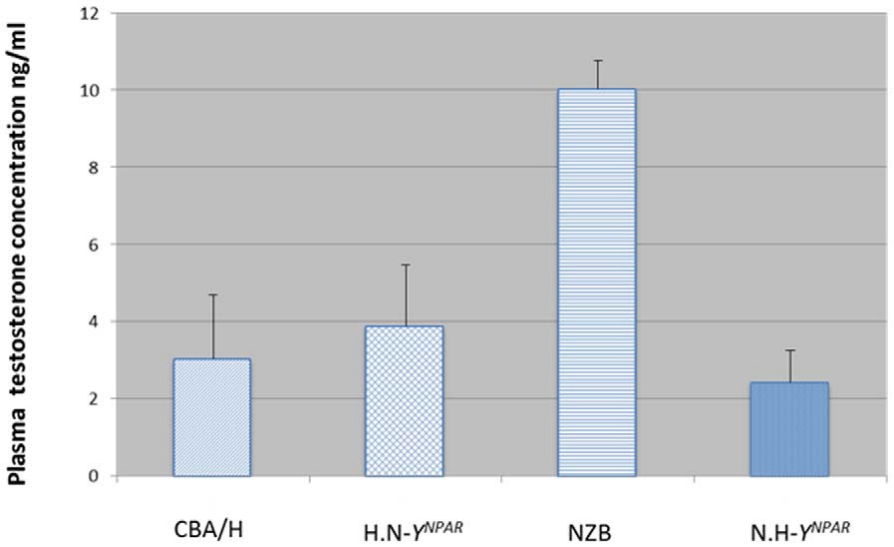
Plasma testosterone concentration (mean ±SEM) in NZB and CBA/H and their consomic strains for *Y^NPAR^*. The N.H-*Y^NPAR^* differs only from the NZB by the *Y^NPAR^* from CBA/H, and the H.N-*Y^NPAR^* differs only from the CBA/H by the *Y^NPAR^* from NZB. Partial comparisons with Student's t test showed that the parental NZB and CBA/H strains differed significantly (*P*<0.0001), and the parental NZB strain differed significantly from its consomic strain (NZB *vs.* N.H-*Y^NPAR^*: *P*<0.001); *n* = 10 animals for each strain; *SEM* = standard error of the mean.

The non-*Y^NPAR^* genotype, the *Y^NPAR^* region and their interactions modulated significantly plasma testosterone concentration (F (1,17) = 48.42, *P*<0.0001, *θ^2^* = 18.20; F(1,17) = 109.15, *P*<0.0001, *θ^2^* = 0.41 and F(1,17) = 72.63, *P*<.0001, *θ^2^* = 27.28, respectively). Partial comparisons indicated that testosterone concentration were significantly higher in the N strain than in the H strain (t (5.99) = 11.97, *P*<0.0001). The substitution of the *Y^NPAR^* from N by the *Y^NPAR^* from H, in the N strain, reduce significantly the testosterone concentration (t (5.99) = 13.37, *P*<0.001), whereas the opposite replacement of the *Y^NPAR^* from H by the *Y^NPAR^* from N, in the H strain, did not modify the testosterone concentration (see **Figure 2**).

### Correlation between β-endorphin and testosterone

The data of brain BE levels and plasma testosterone concentrations (mean ± SEM) in the 11 inbred strains are presented in **Table 1**. The 11 inbred mouse strains did not differ significantly for BE or testosterone concentrations. In addition, there was no correlation between brain BE levels and plasma testosterone concentration when the analysis was conducted on the eleven different inbred strains including the same N and H males contributing to the present study (*ρ* spearman = 0.09, *P*>0.1).

**Table 1:** Brain β-Endorphin and plasma Testosterone concentrations (Mean ± SEM) in 11 inbred mouse strains (*n* = 5).

	A/J	C57BL/10Bg	C57BL/6	BA	BALB/cBy	CBA/H	CPB-K	DBA/1Bg	DBA/2j	NZB	XLII	F Value
**Biological variables**												
β-Endorphin	271.5	403.4	143.8	111.8	191.0	351.0	133.4	361.2	342.6	191.8	99.2	1.31
(pmoles/g)	±35.4	±250.2	±24.6	±8.7	±17.8	±59.1	±32.1	±260.1	±191.4	±37.42	±16.5	
Testosterone	1.9	3.5	1	2.2	1.3	0.23	0.81	0.9	0.58	4.7	0.36	1.25
(ng/ml)	±1.65	±1.61	±0.8	±1.79	±1.16	±0.08	±0.35	±0.31	±0.31	±1.55	±0.22	

*Note:* Comparisons between the 11 strains for β-Endorphin and Testosterone concentrations were performed using ANOVA with F values indicated in the table; *n* = 5 animals for each strain, except for NBZ/BINj (*n* = 6); *SEM* = standard error of the mean.

## Discussion

Our main data indicated a significant and major effect of the *Y^NPAR^* in interaction with the genetic background on brain BE levels. The controls to ensure the isogenicity of the background in each parental strain and its consomic strain rule out a possible contribution of residual autosomal alleles from the donor strain to this effect of the *Y^NPAR^* in interaction with the genetic background. None of the annotations of the genes carried by the *Y^NPAR^* allows us to consider that one of them contributes directly in the BE production [43].

An indirect effect of the *Y^NPAR^* in interaction with the genetic background on brain BE levels via plasma testosterone is not supported by our findings. Indeed, there was no correlation in this study between brain BE levels and plasma testosterone concentrations, although this research is limited by the absence of variability of BE or testosterone concentration between the 11 inbred strains. In addition, our results on plasma testosterone measured in the parental N and H and their consomic strains confirm our previous study [12] and indicate that the strain distribution pattern differs for brain BE levels and plasma testosterone concentrations; the *Y^NPAR^* from N, which depleted BE on the H background, had no effect on testosterone. Thus, the strong decremental effect on brain BE levels resulting from the transfer of the *Y^NPAR^* from N on H, cannot be explained by variations of plasma testosterone concentrations. Furthermore, the statistical effects of the *Y^NPAR^* substitution are not the same in BE and testosterone: first, the *Y^NPAR^* modulates BE concentration via an interactive effect with the genetic background and with no significant effect of *Y^NPAR^* alone, whereas testosterone concentration is modulated by the *Y^NPAR^* substitution alone and by an interaction between the *Y^NPAR^* and the genetic background. Finally, effect size of the non-*Y^NPAR^* genotype, the *Y ^NPAR^* and their interactions differ for BE and testosterone.

The indirect effect of the *Y^NPAR^* in interaction with the genetic background on brain BE levels might be explained by a contribution of the sex-determining region of the Y chromosome (*Sry*) carried by *Y^NPAR^* [44]–[47]. Thus, the transcription of the Melanocortin receptor (*Mc2R*) gene depends on the presence of *Sry* [45], [48]. *Mc2R* gene, located on chromosome 18, has been identified as the gene of the receptor of proopiomelanocortin. Proopiomelanocortin plays an important role in the BE system given that BE is directly derived from proopiomelanocortin. The *Y^NPAR^* has different origins in inbred strains of mice. The N and H strains are from Asian and European origins, respectively. An analysis of the *Y^NPAR^* patterns of restriction reveals that the N and H strains belong to different groups [49] suggesting *Sry* polymorphisms. We hypothesized that *Sry* polymorphisms between *Y^NPAR^* from N and *Y^NPAR^* from H, modify the transcription of several genes including *Mc2R*, resulting in the modification of BE brain concentration.

The effect of the *Y^NPAR^* on brain BE concentration in interaction with the genetic background reported in the present study is the first demonstration of Y-chromosome mediated control of brain opioid. The results open new perspectives to better understand sex differences observed in some disorders such as autistic disorder, where abnormal central BE levels have been reported. Finally, the findings could have important implications for research on the genetic control of BE metabolic pathways.
